# Molecular identification of two *Culex (Culex)* species of the neotropical region (Diptera: Culicidae)

**DOI:** 10.1371/journal.pone.0173052

**Published:** 2017-02-24

**Authors:** Magdalena Laurito, Ana M. Ayala, Walter R. Almirón, Cristina N. Gardenal

**Affiliations:** 1 Instituto de Investigaciones Biológicas y Tecnológicas (IIByT) CONICET-UNC. Centro de Investigaciones Entomológicas de Córdoba (CIEC)—Universidad Nacional de Córdoba, Córdoba, Argentina; 2 Instituto de Diversidad y Ecología Animal (IDEA) CONICET -Universidad Nacional de Córdoba, Córdoba, Argentina; Sichuan University, CHINA

## Abstract

*Culex bidens* and *C*. *interfor*, implicated in arbovirus transmission in Argentina, are sister species, only distinguishable by feature of the male genitalia; however, intermediate specimens of the species in sympatry have been found. Fourth-instar larvae and females of both species share apomorphic features, and this lack of clear distinction creates problems for specific identification. Geometric morphometric traits of these life stages also do not distinguish the species. The aim of the present study was to assess the taxonomic status of *C*. *bidens* and *C*. *interfor* using two mitochondrial genes and to determine the degree of their reproductive isolation using microsatellite loci. Sequences of the ND4 and COI genes were concatenated in a matrix of 993 nucleotides and used for phylogenetic and distance analyses. Bayesian and maximum parsimony inferences showed a well resolved and supported topology, enclosing sequences of individuals of *C*. *bidens* (0.83 BPP, 73 BSV) and *C*. *interfor* (0.98 BPP, 97 BSV) in a strong sister relationship. The mean K2P distance within *C*. *bidens* and *C*. *interfor* was 0.3% and 0.2%, respectively, and the interspecific variation was 2.3%. Bayesian clustering also showed two distinct mitochondrial lineages. All sequenced mosquitoes were successfully identified in accordance with the best close match algorithm. The low genetic distance values obtained indicate that the species diverged quite recently. Most morphologically intermediate specimens of *C*. *bidens* from Córdoba were heterozygous for the microsatellite locus GT51; the significant heterozygote excess observed suggests incomplete reproductive isolation. However, *C*. *bidens* and *C*. *interfor* should be considered good species: the ventral arm of the phallosome of the male genitalia and the ND4 and COI sequences are diagnostic characters.

## Introduction

The genus *Culex* L., with a worldwide distribution and 770 species grouped in 26 subgenera [1], is widely known for its medical and veterinary importance. Among the subgenera, subgenus *Culex* includes 200 species [1], many of them recognized as vectors of filarial nematodes [2] and several arboviruses [3]. The subgenus is not monophyletic [4,5,6] and the infrasubgeneric categories are based on superficial similarities that may not reflect natural relationships [7]. The subgenus exhibits polymorphic features and exceptional forms [8] and is arranged in an informal classification that includes Series, Groups, Subgroups and Complexes, with nine species without placement [1]. Morphological identification of *Culex* (*Culex*) species is difficult because anatomical traits of fourth-instar larvae and females are polymorphic and overlap among species. Only morphological features of the male genitalia provide a wealth of taxonomic characters that allow reliable identification of the species, as well as synapomorphic traits that contribute to revolving phylogenetic relationships in the subgenus [9].

*Culex bidens* Dyar and *C*. *interfor* Dyar are a clear example of the problematic identification described above. The former is widely distributed in many countries of the Neotropical Region from Mexico to Argentina; the latter is restricted to Argentina and Paraguay [10]. The species are grouped in a well-supported clade as sister species on the basis of morphological characters [9]. These species share apomorphic features of fourth-instar larvae and females, being only distinguishable by some male genitalia traits [9]. The two species share features of the subapical lobe of the gonocoxopodites, paraproct and dorsal arms of the phallosome. *Culex bidens* differs by having lateral plate with 1‒3 (usually 2) large dorsolaterally directed teeth, 0‒3 minute conical denticles and the ventral arm developed as a spine bent dorsolaterally. The lateral plate of C. interfor has a single strong tooth directed dorsolaterally (infrequently with denticles), and the ventral arm is small, triangular and laterally directed flap-like process [11]. Besides morphological characters, geometric morphometric procedures were also assessed to distinguish these species from each other and from other species [12], revealing that shapes of wings of adults and dorsomentum of larvae could not distinguish them. Furthermore, intermediate features in male genitalia in sympatric populations of the two species have recently been found, suggesting that successful interbreeding could be occurring in the localities of Resistencia (Chaco Province), Córdoba and Río Seco (Córdoba Province), Puerto Iguazú (Misiones Province) and Chamical (La Rioja Province) of Argentina [13].

Distinguishing closely related species using morphological traits is not easy, even more so if there are intermediate specimens. Accurate identification is of paramount importance to determine areas of potential risk of disease transmission, particularly when dealing with invasive mosquito species that are potential disease vectors. The need to distinguish *C*. *bidens* and *C*. *interfor* is important because they have been implicated in the transmission of two arboviruses. *Culex bidens* was assumed to be the vector of *Venezuelan Equine Encephalitis* virus (VEEV) during the epizootic in Chaco Province in 1988 [14] and *C*. *interfor* is considered a secondary vector of *Saint Louis Encephalitis* virus (SLEV) [15] in Córdoba, Argentina. To achieve a correct identification of these species, alternative tools, such as molecular methods, are needed.

To be effective, a diagnostic molecular marker must demonstrate consistent differences between closely related species [16]. Mitochondrial genes are considered good markers because of their abundance, lack of introns, limited exposure to recombination, and haploid mode of inheritance [17]. Among mitochondrial genes, a fragment of the *cytochrome c oxidase subunit I* (*COI*) has been largely employed for taxon barcoding [18], and to estimate genetic divergence among phylogenetically close species [19]. In mosquitoes, *COI* barcode sequences have been used to successfully identify species [20,21,22] and to recognize species complexes [23,24,25]. Even though Laurito *et al*. [26] revealed that the *COI* barcode fragment does not contain enough information to identify all species of *Culex* (*Culex*) from the Neotropical Region, about 40% of the samples clustered with their conspecifics in that study. Another DNA region, the *nicotinamide adenine dinucleotide dehydrogenase subunit 4* (*ND4*) has recently been employed for specific identification in *Aedes koreicus* (Edwards) populations far away from its known natural range of distribution [27]. Cameron *et al*. [28] designed a molecular assay based on *ND4* sequences to identify species of *Aedes* Meigen.

The lack of appropriate diagnostic morphological characters to identify *C*. *bidens* and *C*. *interfor* in all stages except males, their close phylogenetic relationship, the sympatric occurrence of intermediate specimens and the poor differentiation based on geometric morphometrics lead us to question the taxonomic rank of these species. The aim of the present study was to assess the taxonomic status of *C*. *bidens* and *C*. *interfor* using a mitochondrial DNA-based phylogenetic molecular approach. Additionally, three microsatellite loci were tested to get complementary information based on other than maternally inherited markers, which could provide evidence about the degree of reproductive isolation between these species.

## Material and methods

### Mosquito collection

**Fig 1:**
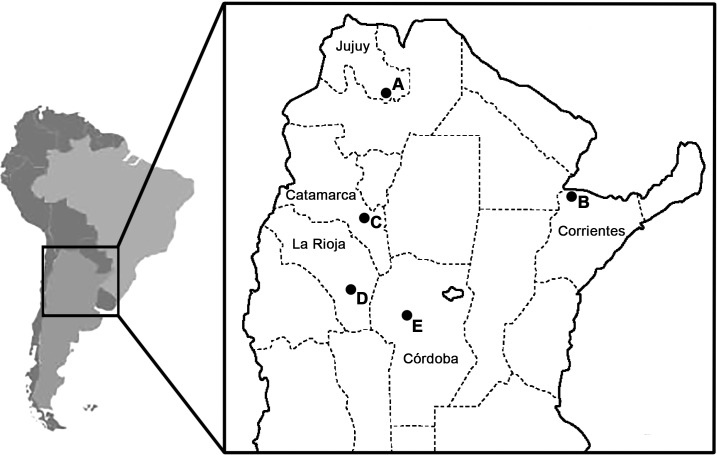
Collection locations in Argentina. A: Puesto Viejo (Jujuy Province); B: National Route 12 (Corrientes Province); C: La Puerta (Catamarca Province); D: Chamical (La Rioja Province); E: Córdoba (Córdoba Province).

Fourth-instar larvae were collected between 2012 and 2015 in the Catamarca (28°11'1.7''S—65°48'40.7''W), Córdoba (31°21'14.26''S—64°06'7.49''´W), Corrientes (27°21'59.07''S—58°16'58.9''W), Jujuy (24°29'11.51"S—64°58'5.50"W) and La Rioja (30°20'45.4"S—66°19'33.4"W) Provinces of Argentina (Fig 1). Capture of specimens and their transport to the laboratory for subsequent identification were permitted by Secretaría de Ambiente (La Rioja Province), Secretaría de Estado del Ambiente y Desarrollo Sustentable (Catamarca Province), Secretaría de Ambiente y Cambio Climático (Córdoba Province), Ministerio de Ambiente (Jujuy Province) and Secretaría de ambiente y Desarrollo Sustentable (Corrientes Province). Field studies did not involve endangered or protected species. Larvae were individually reared and 17 males of each species were identified based on features of their genitalia [11] (S1 Table). Five sequences of *C*. *bidens* from Córdoba (Cba1507, Cba1514, Cba1526, Cba1532 and Cba1547) and one of *C*. *interfor* (Cba1505) corresponded to specimens which showed intermediate features of the male genitalia, similar to those described in Laurito and Almirón [13]. Fourth-instar larval and pupal exuviae and mounted male genitalia stored as vouchers are deposited in the Entomological Collection of the Centro de Investigaciones Entomológicas de Córdoba (Universidad Nacional de Córdoba).

### Molecular procedures

Genomic DNA from each specimen was obtained from one or two legs preserved in absolute alcohol, using 60 μL of Chelex-100 5% (w/v) and 30 μL of NaCl 0.9% (w/v). The extract solution was vortexed and incubated at 100°C for 15 min. After centrifugation at 13,000 rpm for 10 min at room temperature, 30–50 μL of supernatant was recovered. The DNA concentration was determined by comparison with known amounts of electrophoretic standards (λ/*Hind* III, Promega, USA), run on 0.8% agarose gels with ethidium bromide. DNA was eluted to a final concentration of 10 ng/μl and stored at -20°C. *ND4* (~336 bp) and *COI* (~658 bp) genes were amplified as in Da Costa-da-Silva *et al*. [29] and Folmer *et al*. [30], respectively, with minor modifications. The PCR products were electrophoresed in 1% agarose gels-TBE buffer and stained with ethidium bromide. All sequencing reactions were carried out in both directions using an ABI3730XL automatic sequencer (Macrogen Inc., Korea) with the same set of PCR primers.

A panel of 12 microsatellite loci developed for other *Culex* (*Culex*) species (*C*. *pipiens* L. and *C*. *quinquefasciatus* Say) were screened, but only three were successfully amplified for *C*. *bidens* and *C*. *interfor*. The PCR reactions were performed as described in Fonseca *et al*. [31] using primers CQ41 and CQ11, and in Keyghobadi *et al*. [32] using primer GT51. The PCR products were visualized on 8% polyacrylamide gels stained with silver nitrate [33] and sized by comparison with a marker of 10 pb (Invitrogen, Argentina), using electrophoresis.

### Mitochondrial and microsatellite data analyses

The sequences for each mitochondrial gene were edited using BioEdit v. 7.2 [34]; primer regions were removed. Comparisons with available sequences using Basic Local Alignment Search Tool (blast.ncbi.nlm.nih.gov/Blast.cgi) were performed to check for sequence homology. Sequences were aligned by nucleotides using the Muscle algorithm [35] in SeaView v. 4 [36] and by amino acids using TranslatorX [37]. Aligned nucleotide sequences of the *ND4* and *COI* genes were concatenated in a single data matrix using Mesquite [38]. Haplotype (Hd) and nucleotide (π) diversities for each gene and species were calculated using DnaSP v5 [39]. Allele frequencies at each microsatellite locus were calculated only for populations of Córdoba and La Rioja since very low sample size of both species was obtained in Catamarca; in Jujuy and Corrientes, only one of the species, *C*. *bidens* and *C*. *interfor* respectively, was collected (S1 Table).

### Phylogenetic inference

The best partitioning scheme and corresponding nucleotide substitution models were selected with the program PartitionFinder v. 1.0.1 [40], using the Bayesian Information Criterion (BIC). Bayesian phylogenetic (BP) analysis was conducted in MrBayes v. 3.1.2 [41] for the combined data matrix. Six independent runs of simultaneous MCMC (Markov Chain Monte Carlo) chains (temperature = 0.15) were performed for 2.5 million generations. Trees were sampled every 2,000 generations with the first 250 trees discarded as burn-in. The standard deviation of the split frequencies between runs (<0.01) and the effective sample size (ESS), were monitored to ensure stability, convergence and correct mixing of the chains.

Maximum parsimony (MP) analyses implemented in TNT v. 1.5 [42] using equal weighting and gaps treated as missing data were conducted to corroborate the topology of the BP results. The search for Wagner trees was performed using a series of 1,000 random addition sequences, retaining up to 100 trees per replication, and TBR as branch rearrangements followed by a second heuristic search. Statistical support for groups was estimated using bootstrap values (BSV) obtained from 1,000 bootstrap replicates. Kimura two-parameter (K2P) distances between and within *C*. *bidens* and *C*. *interfor* were estimated with Mega v. 6 [43]. Sequences of both genes of three other species of the subgenus *Culex* recorded for Argentina, *C*. *coronator* Dyar and Knab (KY581244/KY581243), *C*. *pipiens* (NC015079) and *C*. *quinquefasciatus* (NC014574), were obtained from GenBank and included in the analyses. *Anopheles darlingi* Root (NC014274) was used as outgroup.

### *ND4* + *COI* for species identification

The usefulness of the *ND4* + *COI* genes for species identification was tested following the best close match (BCM) criterion developed by Meier *et al*. [44]. The algorithm identifies the best sequence matches of a query and only assigns the species name of that sequence to the query if the sequence is sufficiently similar. To determine how similar the sequences are, a threshold similarity value was estimated for the dataset by obtaining a frequency distribution of all intraspecific pairwise distances and determining the distance below which 95% of all intraspecific distances were found. The best sequence matches and the mentioned threshold were estimated using TaxonDNA (taxondna.sf.net/). Queries without sequence match below the threshold value remain unidentified. In contrast, those queries with match above the threshold value were considered a successful, ambiguous or incorrect identification. A correct identification was achieved if both names are identical. When at least two equally good best matches were found the identification remained ambiguous and when the names were mismatched, the identification was a failure.

## Results

### Mitochondrial sequences

Phylogenetic inferences, K2P distances between and within species and the BCM algorithm were performed for each gene separately and combined. Since the outcomes of analyzing *ND4* and *COI* separately and in combination remained unchanged, only results obtained with the latter are shown. The alignment of *ND4* and *COI* was trivial because they showed no evidence of insertion/deletion events. The total length of the 34 concatenated aligned data matrix was 993 nucleotides (336 bp of *ND4* and 657 bp of *COI*). After alignment, 17 sequences were recovered in DAMBE v. 5.3.2 [45].

Five haplotypes of the *ND4* fragment were found in *C*. *bidens*, and five in *C*. *interfor*, with genetic diversity indexes relatively high in both species, with Hd = 0.757 (π = 0.00326) and Hd = 0.742 (π = 0.00315), respectively. Six haplotypes of the *COI* fragment were found in *C*. *bidens* and four in *C*. *interfor*. Genetic diversity in this fragment was, in both species, lower than that observed in *ND4*, being Hd = 0.702 (π = 0.003) in *C*. *bidens* and Hd = 0.618, (π = 0.00118) in *C*. *interfor*.

### Microsatellite loci

Locus GT51 showed two alleles, allele 90 being predominant in *C*. *bidens* and allele 88 in *C*. *interfor*. In the latter species, allele 90 was not detected in the specimens from Córdoba and La Rioja but it was present in one of three individuals from Catamarca and in one of three from Corrientes. Four of the five specimens from Córdoba showing intermediate features in male genitalia but belonging to the *C*. *bidens* lineage of mtDNA, were heterozygous 88/90 in locus GT51; the other specimen was homozygous 90/90. Loci CQ41 and CQ11 exhibited five and six alleles, respectively. In CQ11, alleles 82 and 208 were detected only in *C*. *bidens* (Table 1). Individual genotypes at the three microsatellite loci are shown in S2 Table.

**Table 1:** Summary of information for the three microsatellite loci isolated in the study. Sample sizes are provided in parentheses.

Locus	Allele	*C*. *bidens*		*C*. *interfor*	
		Cba (12)	LR (3)	Cba (5)	LR (5)
		Frequency			
**GT51**	88	0.36	0.17	1	1
	90	0.64	0.83	0	0
**CQ41**	162	0.04	0	0.3	0
	164	0.04	0	0.4	0.7
	166	0.27	0	0	0.1
	168	0.41	0.83	0.1	0.1
	174	0.23	0.17	0.2	0.1
**CQ11**	82	0.1	0	0	0
	124	0.2	0.17	0.1	0.17
	148	0.6	0.67	0.5	0.83
	176	0.05	0	0.2	0
	202	0	0	0.2	0
	208	0.05	0.17	0	0

Cba: Córdoba. LR: La Rioja.

### Phylogenetic inference

**Fig 2:**
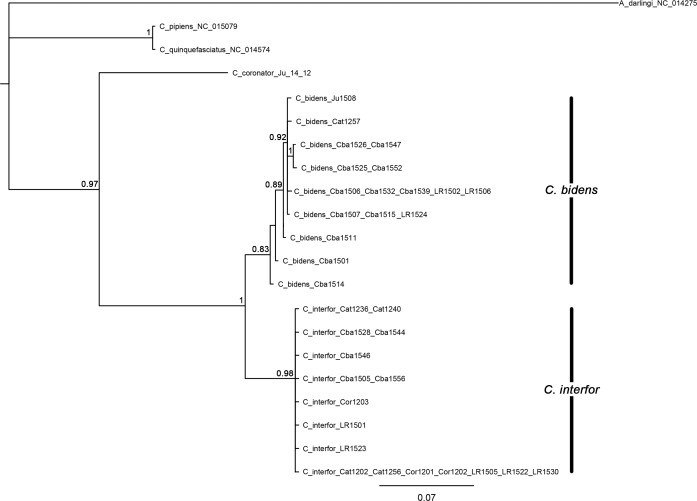
Bayesian tree of combined *ND4* and *COI* sequences from *C*. *bidens* and *C*. *interfor*. The data were partitioned according to the best-fit partitioning schemes selected by the BIC in PartitionFinder. Numbers at branches indicate Bayesian posterior probabilities (≥ 70%). *Anopheles darlingi* was included as outgroup.

Because the topologies of the Bayesian and maximum parsimony trees were consistent and the clades were strongly supported in both analyses, only the former is shown in Fig 2. The best-fit partitioning schemes and models of molecular evolution for each partition selected by the BIC in PartitionFinder are as follows: for *ND4* position 1/*COI* position 2 partition, TrN [46] evolution model was selected; for *ND4* position 2/ *COI* position 3, F81 [47] was the preferred model; for *ND4* position 3/ *COI* position 1, the HKI + G (gamma distribution) [48] model was chosen. Results of Bayesian analysis (Fig 2) showed a well resolved (Bayesian Posterior Probability, BPP) topology. Both clades enclosing sequences of individuals of *C*. *bidens* and *C*. *interfor* were strongly supported, 0.83 BPP (73 BSV) and 0.98 BPP (97 BSV), respectively, as well as their sister relationship (1 BPP, 100 BSV). The five sequences of *C*. *bidens* and the one of *C*. *interfor* corresponding to individuals with intermediate features of male genitalia grouped within the clade representing each of the species, in accordance with their morphological identification. *Culex coronator* was recovered as the sister species (0.97 BPP, 100 BSV) to the *C*. *bidens* + *C*. *interfor* clade (Fig 2).

The pairwise K2P distances between the 34 sequences are shown in S3 Table. The genetic divergence within *C*. *bidens* varied from 0–0.91% (mean: 0.3%), and in *C*. *interfor* between 0–0.3% (mean: 0.2%) while the mean interspecific variation was 2.30% (ranging from 1.94 to 2.56%). The interspecific distance values were 7.67 and 11.50 times higher than the variability within *C*. *bidens* and *C*. *interfor*, respectively.

### *ND4* + *COI* for species identification

Ninety-five percent of the intraspecific K2P distances were found within the interval between 0–2.5%; the higher value was used as cut-off to define the limit for species identification. All sequenced mosquitoes were successfully identified in accordance with the BCM (S4 Table).

## Discussion

Differentiation of *C*. *bidens* and *C*. *interfor* is an example of problematic identification in the subgenus *Culex* because they share apomorphic features in the fourth-instar larvae and females [12]. As mentioned above, they are sister species only distinguishable by some male genitalia traits [11]. However, the finding of intermediate specimens for these characters in sympatric populations [13] poses doubts about their specific status. In these cases, the use of molecular tools can help to assess taxonomic units and to infer the possible existence of hybridization between species.

The two mitochondrial genes here assayed revealed moderate haplotype diversity in *C*. *bidens* and *C*. *interfor* (for *ND4* and *COI*, mean Hd = 0.749 and 0.66, respectively), with most haplotypes within each species differing by a single mutation. Intraspecific nucleotide diversity at both genes was relatively high (15 and 16 polymorphic sites in *C*. *bidens* and *C*. *interfor*, respectively). Using *ND4* as a molecular marker, Venkatesan *et al*. [49] recorded an Hd value ranging from 0.775 to 0.962 among eight populations of *C*. *tarsalis* Coquillett from western United States, and Morais *et al*. [50] obtained a unique haplotype of *C*. *quinquefasciatus* from nine localities of Brazil. For the *COI* region, Pfeiler *et al*. [51] reported an Hd of 0.921 and 0.071 in populations of *C*. *tarsalis* and *C*. *quinquefasciatus*, respectively, from the Sonoran Desert (North America), while Morais *et al*. [50] obtained an Hd = 0.636 in *C*. *quinquefasciatus* from Brazil.

The two species considered here do not share any haplotype of mitochondrial DNA. The Bayesian and the maximum parsimony phylogenetic inferences recovered two strongly supported clades, each containing sequences corresponding to individuals classified as either *C*. *bidens* or *C*. *interfor*. According to our results, the two species represent monophyletic maternal lineages based on sequences of the *ND4* and *COI* genes, considered separately as well as in a concatenated analysis. Other molecular phylogenetic approaches also demonstrated the robustness of DNA markers to identify *Culex* species [52,5,26], *Aedes* subspecies [27] and complexes in the genera *Culex* [53,54] and *Anopheles* [23,24,25]. Hoyos-López *et al*. [55] used DNA from legs of female mosquitoes to amplify the *COI* barcode region as a method to corroborate the taxonomic identity of pools of *C*. *erraticus* (Dyar and Knab) and *Mansonia titillans* (Walker) that were found positive for arboviruses.

Ruiz-Lopez *et al*. [23] poseded that intraspecific distance in mosquitoes may vary from 0–2%, which is in agreement with our observations for *C*. *bidens* and *C*. *interfor*. Hebert *et al*. [56] proposed that the divergence of the closest species should be at least 10 times higher than the average intraspecific genetic distance. In our study, interspecific distance value only agreed with this proposal for *C*. *interfor* (11.5 times higher than intraspecific distance); in *C*. *bidens* the rate was below 10. The low levels of interspecific divergence suggest a recent separation of the two lineages. However, the two genetic markers here employed allowed us to unequivocally identify all the sequences analyzed as belonging to either *C*. *bidens* or *C*. *interfor* (S4 Table).

The six specimens exhibiting intermediate traits of the male genitalia sequenced in this study and morphologically similar to those described in Laurito and Almirón [13], led us to restrict the diagnostic features to the shape of the ventral arm of the phallosome as a follows: *C*. *bidens* has the ventral arm developed as a spine bent dorsolaterally and *C*. *interfor* has the ventral arm developed as a small, triangular, laterally directed flap-like process. This character corresponded fully with the *ND4* and *COI* mtDNA markers, both separately and in combination, which intermediate forms to be assigned to one or other of the two species, using both morphological and molecular criteria.

Because of their co-dominance, microsatellites proved to be useful for detecting putative hybrids between closely related species and subspecies of *Culex* [57,58,59]. In the present study, the main difficulty in applying this methodology was the lack of species-specific primers for PCR reactions, and for this reason only three loci could be amplified using primers developed for other species of the genus. Also, a few morphologically intermediate specimens in the cities of Córdoba and La Rioja were collected in spite of an extensive sampling effort. However, several interesting observations emerge from our data. No single allele at any loci could be assigned to one or other of the two species. Loci CQ11 and CQ41 did not show significant differences in allele frequencies between the species, and although alleles 82 and 208 in locus CQ11 were only detected in *C*. *bidens*, given the low sample sizes analyzed they should not be considered as exclusive. In locus GT51, allele 90 was dominant in *C*. *bidens* and allele 88 was the only one present in specimens of *C*. *interfor* from Córdoba and La Rioja (Table 1), although it was detected in specimens from other sites. These results indicate that at least the nuclear markers here assayed are not useful for distinguishing *C*. *bidens* from *C*. *interfor*. On the other hand, it is worth noting that four of five specimens from Córdoba, belonging to the *C*. *bidens* mitochondrial lineage but exhibiting intermediate male genitalia traits, were heterozygous 88/90, supporting the concept of incomplete reproductive isolation between *C*. *interfor* and *C*. *bidens*. It is also important to note that immature stages of the intermediate specimens analyzed were collected from the same habitat with both parental species. A sympatric distribution of *C*. *bidens* and *C*. *interfor* larvae was also observed in populations from other sampling sites. Considering the available morphological data, biotic sympatry and molecular biparental markers, some degree of hybridization between these species seems plausible. However, greater sample sizes and the use of species-specific primers for microsatellite loci are needed to analyze their genetic population structure and to distinguish between purebred and hybrid individuals of different generations, employing Bayesian methods.

Results presented here confirm the status of *C*. *bidens* and *C*. *interfor* as species, the ventral arm of the phallosome and *ND4* or *COI* gene sequences being diagnostic characters for their accurate identification. However, studies based on a population genetics approach will be required to obtain a fuller understanding about the degree and mechanisms of isolation between these sympatric species.

## Supporting information

S1 Table Information for male specimens included in the study, including specimen identification codes, collection localities, geographical coordinates, collector and GenBank accession numbers. (PDF)

S2 Table Individual genotypes at the three microsatellite loci analyzed. *: morphologically intermediate specimens.
(PDF)

S3 Table K2P distances between 34 concatenated ND4 + COI sequences of *C*. *bidens* and *C*. *interfor*. (PDF)

S4 Table Identification of *C*. *bidens* and *C*. *interfor* based on best close match employing ND4 + COI concatenated sequences. (PDF)
