# Supplementary material for: Molecular identification of two Culex (Culex) species of the neotropical region (Diptera: Culicidae)
Source: PLoS One. 2017 Feb 24;12(2):e0173052. doi: 10.1371/journal.pone.0173052 (PMC5325596; doi:10.1371/journal.pone.0173052)
Supplement: S1 Table — (PDF) [file pone.0173052.s001.pdf]

Species	Sample ID	Locality	Province	Latitude	Longitude	Collector	GenBank ND4 / COI
C. bidens	Cat1257	LP	Cat	28º11'1.7"S	65º48'40.7"W	ML-WRA	KY581210/KY581209
C. bidens	Cba1501	Cba	Cba	31°21'14.26"S	64°06'7.49"´W	ML-WRA	KY581223/KY581211
C. bidens	Cba1506	Cba	Cba	31°21'14.26"S	64°06'7.49"´W	ML-WRA	KY581224/KY581212
C. bidens	Cba1507*	Cba	Cba	31°21'14.26"S	64°06'7.49"´W	ML-WRA	KY581225/KY581213
C. bidens	Cba1511	Cba	Cba	31°21'14.26"S	64°06'7.49"´W	ML-WRA	KY581226/KY581214
C. bidens	Cba1514*	Cba	Cba	31°21'14.26"S	64°06'7.49"´W	ML-WRA	KY581227/KY581215
C. bidens	Cba1515	Cba	Cba	31°21'14.26"S	64°06'7.49"´W	ML-WRA	KY581228/KY581216
C. bidens	Cba1525	Cba	Cba	31°21'14.26"S	64°06'7.49"´W	ML-WRA	KY581229/KY581217
C. bidens	Cba1526*	Cba	Cba	31°21'14.26"S	64°06'7.49"´W	ML-WRA	KY581230/KY581218
C. bidens	Cba1532*	Cba	Cba	31°21'14.26"S	64°06'7.49"´W	ML-WRA	KY581231/KY581219
C. bidens	Cba1539	Cba	Cba	31°21'14.26"S	64°06'7.49"´W	ML-WRA	KY581232/KY581220
C. bidens	Cba1547*	Cba	Cba	31°21'14.26"S	64°06'7.49"´W	ML-WRA	KY581233/KY581221
C. bidens	Cba1552	Cba	Cba	31°21'14.26"S	64°06'7.49"´W	ML-WRA	KY581234/KY581222
C. bidens	Ju1508	PV	Ju	24°29'11.51"S	64°58'5.50"W	MAL	KY581236/KY581235
C. bidens	LR1502	Cha	LR	30°20'45.4"S	66°19'33.4"W	AMV-ML	KY581240/KY581237
C. bidens	LR1506	Cha	LR	30°20'45.4"S	66°19'33.4"W	AMV-ML	KY581241/KY581238
C. bidens	LR1524	Cha	LR	30°20'45.4"S	66°19'33.4"W	AMV-ML	KY581242/KY581239
C. interfor	Cat1202	LP	Cat	28º11'1.7"S	65º48'40.7"W	AMV-ML	KY581249/KY581245
C. interfor	Cat1236	LP	Cat	28º11'1.7"S	65º48'40.7"W	AMV-ML	KY581250/KY581246
C. interfor	Cat1240	LP	Cat	28º11'1.7"S	65º48'40.7"W	AMV-ML	KY581251/KY581247
C. interfor	Cat1256	LP	Cat	28º11'1.7"S	65º48'40.7"W	AMV-ML	KY581252/KY581248
C. interfor	Cba1505*	Cba	Cba	31°21'14.26"S	64°06'7.49"´W	ML-WRA	KY581258/KY581253
C. interfor	Cba1528	Cba	Cba	31°21'14.26"S	64°06'7.49"´W	ML-WRA	KY581259/KY581254
C. interfor	Cba1544	Cba	Cba	31°21'14.26"S	64°06'7.49"´W	ML-WRA	KY581260/KY581255
C. interfor	Cba1546	Cba	Cba	31°21'14.26"S	64°06'7.49"´W	ML-WRA	KY581261/KY581256
C. interfor	Cba1556	Cba	Cba	31°21'14.26"S	64°06'7.49"´W	ML-WRA	KY581262/KY581257
C. interfor	Cor1201	NR12	Cor	27º21'59.07"S	58°16'58.9"W	ML	KY581266/KY581263
C. interfor	Cor1202	NR12	Cor	27º21'59.07"S	58°16'58.9"W	ML	KY581267/KY581264
C. interfor	Cor1203	NR12	Cor	27º21'59.07"S	58°16'58.9"W	ML	KY581268/KY581265
C. interfor	LR1501	Cha	LR	30°20'45.4"S	66°19'33.4"W	AMV-ML	KY581274/KY581269
C. interfor	LR1505	Cha	LR	30°20'45.4"S	66°19'33.4"W	AMV-ML	KY581275/KY581270
C. interfor	LR1522	Cha	LR	30°20'45.4"S	66°19'33.4"W	AMV-ML	KY581276/KY581271
C. interfor	LR1523	Cha	LR	30°20'45.4"S	66°19'33.4"W	AMV-ML	KY581277/KY581272
C. interfor	LR1530	Cha	LR	30°20'45.4"S	66°19'33.4"W	AMV-ML	KY581278/KY581273

AMV: AM Visintín; Cat: Catamarca; Cba: Córdoba; Cha: Chamical; Cor: Corrientes; MAL: MA Linares; Ju: Jujuy; LP: La Puerta; LR: La Rioja; ML: M Laurito; NR12: National Route 12; PV: Puesto Viejo; WRA: WR Almirón.

*: morphologically intermediate specimens.
