# Supplementary material for: Molecular identification of two Culex (Culex) species of the neotropical region (Diptera: Culicidae)
Source: PLoS One. 2017 Feb 24;12(2):e0173052. doi: 10.1371/journal.pone.0173052 (PMC5325596; doi:10.1371/journal.pone.0173052)
Supplement: S2 Table — *: morphologically intermediate specimens. (PDF) [file pone.0173052.s002.pdf]

Sample	Locus					
	GT51		CQ41		CQ11	
C. bidens Ju1508	88	90	168	168	184	184
C. bidens Cat1257	88	88	168	168	154	202
C. interfor Cat1202	90	90	166	166	202	202
C. interfor Cat1236	88	88	164	166	--	--
C. interfor Cat1240	--	--	164	164	126	158
C. interfor Cat1256	88	88	166	166	158	158
C. bidens Cba1501	90	90	166	166	--	--
C. bidens Cba1506	--	--	166	166	148	148
C. bidens Cba1515	88	90	162	168	148	148
C. bidens Cba1525	88	90	164	168	82	82
C. bidens Cba1539	90	90	166	166	148	148
C. bidens Cba1552	88	90	168	174	148	148
C. bidens Cba1511	88	90	168	168	124	176
C. bidens Cba1507*	90	90	168	168	124	124
C. bidens Cba1526*	88	90	174	174	--	--
C. bidens- ba1532*	88	90	168	168	148	148
C. bidens Cba1547*	88	90	174	174	148	148
C. bidens Cba1514*	88	90	166	166	124	208
C. interfor Cba1505*	88	88	162	162	176	176
C. interfor Cba1528	88	88	164	164	148	148
C. interfor Cba1544	88	88	164	174	148	148
C. interfor Cba1546	88	88	164	174	124	148
C. interfo -Cba1556	88	88	162	168	202	202
C. interfor Cor1201	88	88	162	168	--	--
C. interfor Cor1202	90	90	162	164	158	158
C. interfor Cor1203	88	88	162	162	202	202
C. bidens LR1502	90	90	168	168	148	208
C. bidens LR1506	90	90	168	174	148	148
C. bidens LR1524	88	90	168	168	124	148
C. interfor LR1501	88	88	164	174	148	148
C. interfor LR1505	88	88	164	164	124	148
C. interfor LR1522	88	88	164	164	--	--
C. interfor LR1523	88	88	164	164	148	148
C. interfor LR1530	88	88	166	168	--	--

--: missing data
