# Supplementary material for: Molecular identification of two Culex (Culex) species of the neotropical region (Diptera: Culicidae)
Source: PLoS One. 2017 Feb 24;12(2):e0173052. doi: 10.1371/journal.pone.0173052 (PMC5325596; doi:10.1371/journal.pone.0173052)
Supplement: S3 Table — (PDF) [file pone.0173052.s003.pdf]

	1	2	3	4	5	6	7	8	9	10	11	12	13	14	15	16	17	18
1.*C. bidens* Ju1508																		
2.*C. bidens* Cat1257	0.20																	
3.*C. bidens* Cba1501	0.51	0.30																
4.*C. bidens* Cba1506	0.20	0.20	0.51															
5C. bidens Cba1507	0.10	0.10	0.40	0.10														
6.*C. bidens* Cba1511	0.20	0.20	0.30	0.20	0.10													
7.*C. bidens* Cba1514	0.71	0.51	0.20	0.71	0.61	0.51												
8.*C. bidens* Cba1515	0.10	0.10	0.40	0.10	0.00	0.10	0.61											
9.*C. bidens* Cba1525	0.40	0.40	0.71	0.40	0.30	0.40	0.91	0.30										
10.*C. bidens* Cba1526	0.30	0.30	0.61	0.30	0.20	0.30	0.81	0.20	0.10									
11.*C. bidens* Cba1532	0.20	0.20	0.51	0.00	0.10	0.20	0.71	0.10	0.40	0.30								
12.*C. bidens* Cba1539	0.20	0.20	0.51	0.00	0.10	0.20	0.71	0.10	0.40	0.30	0.00							
13.*C. bidens* Cba1547	0.30	0.30	0.61	0.30	0.20	0.30	0.81	0.20	0.10	0.00	0.30	0.30						
14.*C. bidens* Cba1552	0.40	0.40	0.71	0.40	0.30	0.40	0.91	0.30	0.00	0.10	0.40	0.40	0.10					
15.*C. bidens* LR1502	0.20	0.20	0.51	0.00	0.10	0.20	0.71	0.10	0.40	0.30	0.00	0.00	0.30	0.40				
16.*C. bidens* LR1506	0.20	0.20	0.51	0.00	0.10	0.20	0.71	0.10	0.40	0.30	0.00	0.00	0.30	0.40	0.00			
17.*C. bidens* LR1524	0.10	0.10	0.40	0.10	0.00	0.10	0.61	0.00	0.30	0.20	0.10	0.10	0.20	0.30	0.10	0.10		
18.*C. interfor* Cat1202	2.36	2.25	2.15	2.36	2.25	2.15	2.15	2.25	2.46	2.46	2.36	2.36	2.46	2.46	2.36	2.36	2.25	
19.*C. interfor* Cat1236	2.25	2.15	2.04	2.25	2.15	2.04	2.04	2.15	2.36	2.36	2.25	2.25	2.36	2.36	2.25	2.25	2.15	0.10
20.*C. interfor* Cat1240	2.25	2.15	2.04	2.25	2.15	2.04	2.04	2.15	2.36	2.36	2.25	2.25	2.36	2.36	2.25	2.25	2.15	0.10
21.*C. interfor* Cat1256	2.36	2.25	2.15	2.36	2.25	2.15	2.15	2.25	2.46	2.46	2.36	2.36	2.46	2.46	2.36	2.36	2.25	0.00
22.*C. interfor* Cba1505	2.15	2.04	1.94	2.15	2.04	1.94	1.94	2.04	2.25	2.25	2.15	2.15	2.25	2.25	2.15	2.15	2.04	0.20
23.*C. interfor* Cba1528	2.36	2.25	2.15	2.36	2.25	2.15	2.15	2.25	2.46	2.46	2.36	2.36	2.46	2.46	2.36	2.36	2.25	0.20
24.*C. interfor* Cba1544	2.36	2.25	2.15	2.36	2.25	2.15	2.15	2.25	2.46	2.46	2.36	2.36	2.46	2.46	2.36	2.36	2.25	0.20
25.*C. interfor* Cba1546	2.46	2.36	2.25	2.46	2.36	2.25	2.25	2.36	2.56	2.56	2.46	2.46	2.56	2.56	2.46	2.46	2.36	0.10
26.*C. interfor* Cba1556	2.15	2.04	1.94	2.15	2.04	1.94	1.94	2.04	2.25	2.25	2.15	2.15	2.25	2.25	2.15	2.15	2.04	0.20
27.*C. interfor* Cor1201	2.36	2.25	2.15	2.36	2.25	2.15	2.15	2.25	2.46	2.46	2.36	2.36	2.46	2.46	2.36	2.36	2.25	0.00
28.*C. interfor* Cor1202	2.36	2.25	2.15	2.36	2.25	2.15	2.15	2.25	2.46	2.46	2.36	2.36	2.46	2.46	2.36	2.36	2.25	0.00
29.*C. interfor* Cor1203	2.36	2.25	2.15	2.36	2.25	2.15	2.15	2.25	2.46	2.46	2.36	2.36	2.46	2.46	2.36	2.36	2.25	0.20
30.*C. interfor* LR1501	2.25	2.15	2.04	2.25	2.15	2.04	2.04	2.15	2.35	2.35	2.25	2.25	2.35	2.35	2.25	2.25	2.15	0.20
31.*C. interfor* LR1505	2.36	2.25	2.15	2.36	2.25	2.15	2.15	2.25	2.46	2.46	2.36	2.36	2.46	2.46	2.36	2.36	2.25	0.00
32.*C. interfor* LR1522	2.36	2.25	2.15	2.36	2.25	2.15	2.15	2.25	2.46	2.46	2.36	2.36	2.46	2.46	2.36	2.36	2.25	0.00
33.*C. interfor* LR1523	2.25	2.15	2.04	2.25	2.15	2.04	2.04	2.15	2.36	2.36	2.25	2.25	2.36	2.36	2.25	2.25	2.15	0.10
34.*C. interfor* LR1530	2.36	2.25	2.15	2.36	2.25	2.15	2.15	2.25	2.46	2.46	2.36	2.36	2.46	2.46	2.36	2.36	2.25	0.00

	19	20	21	22	23	24	25	26	27	28	29	30	31	32	33	34
19.*C. interfor* Cat1236	0.20															
20.*C. interfor* Cat1240	0.20	0.00														
21.*C. interfor* Cat1256	0.10	0.10	0.10													
22.*C. interfor* Cba1505	0.30	0.30	0.30	0.20												
23.*C. interfor* Cba1528	0.30	0.30	0.30	0.20	0.20											
24.*C. interfor* Cba1544	0.30	0.30	0.30	0.20	0.20	0.00										
25.*C. interfor* Cba1546	0.20	0.20	0.20	0.10	0.30	0.30	0.30									
26.*C. interfor* Cba1556	0.30	0.30	0.30	0.20	0.00	0.20	0.20	0.30								
27.*C. interfor* Cor1201	0.10	0.10	0.10	0.00	0.20	0.20	0.20	0.10	0.20							
28.*C. interfor* Cor1202	0.10	0.10	0.10	0.00	0.20	0.20	0.20	0.10	0.20	0.00						
29.*C. interfor* Cor1203	0.30	0.30	0.30	0.20	0.20	0.20	0.20	0.30	0.20	0.20	0.20					
30.*C. interfor* LR1501	0.20	0.10	0.20	0.30	0.30	0.30	0.30	0.30	0.30	0.20	0.20	0.30				
31.*C. interfor* LR1505	0.10	0.10	0.10	0.00	0.20	0.20	0.20	0.10	0.20	0.00	0.00	0.20	0,20			
32.*C. interfor* LR1522	0.10	0.10	0.10	0.00	0.20	0.20	0.20	0.10	0.20	0.00	0.00	0.20	0,20	0.00		
33.*C. interfor* LR1523	0.20	0.20	0.20	0.10	0.10	0.10	0.10	0.20	0.10	0.10	0.10	0.10	0,20	0.10	0.10	
34.*C. interfor* LR1530	0.10	0.10	0.10	0.00	0.20	0.20	0.20	0.10	0.20	0.00	0.00	0.20	0,20	0.00	0.00	0.10
