# Supplementary material for: Molecular identification of two Culex (Culex) species of the neotropical region (Diptera: Culicidae)
Source: PLoS One. 2017 Feb 24;12(2):e0173052. doi: 10.1371/journal.pone.0173052 (PMC5325596; doi:10.1371/journal.pone.0173052)
Supplement: S4 Table — (PDF) [file pone.0173052.s004.pdf]

Query	Identification
C. bidens Cat1257	*C. bidens* Cba1501
C. bidens Cba1501	*C. bidens* Cba1514
C. bidens Cba1506	*C. bidens* Cba1532
C. bidens Cba1507	*C. bidens*LR1534
C. bidens Cba1511	*C. bidens* Cba1507
C. bidens Cba1514	*C. bidens* Cba1501
C. bidens Cba1515	*C. bidens* Cba1507
C. bidens Cba1525	*C. bidens* Cba1552
C. bidens Cba1526	*C. bidens* Cba1547
C. bidens Cba1532	*C. bidens* Cba1506
C. bidens Cba1539	*C. bidens* Cba1506
C. bidens Cba1547	*C. bidens* Cba1526
C. bidens Cba1552	*C. bidens* Cba1525
C. bidens Ju1508	*C. bidens* Cba1507
C. bidens LR1502	*C. bidens* Cba1506
C. bidens LR1506	*C. bidens* Cba1506
C. bidens LR1524	*C. bidens* Cba1507
C. interfor Cat1202	*C. interfor* Cat1256
C. interfor Cat1236	*C. interfor* Cat1240
C. interfor Cat1240	*C. interfor* Cat1236
C. interfor Cat1256	*C. interfor* Cor1201
C. interfor Cba1505	*C. interfor* Cba1556
C. interfor Cba1528	*C. interfor* Cba1544
C. interfor Cba1544	*C. interfor* Cba1528
C. interfor Cba1546	*C. interfor* Cat1256
C. interfor Cba1556	*C. interfor* Cba1505
C. interfor Cor1201	*C. interfor* Cat1256
C. interfor Cor1202	*C. interfor* Cat1256
C. interfor Cor1203	*C. interfor* Cat1256
C. interfor LR1501	*C. interfor* Cat1236
C. interfor LR1505	*C. interfor* Cat1256
C. interfor LR1522	*C. interfor* Cat1256
C. interfor LR1523	*C. interfor* Cat1256
C. interfor LR1530	*C. interfor* Cat1256
